# Point‐Combination Transect (PCT): Incorporation of small underwater cameras to study fish communities

**DOI:** 10.1111/2041-210X.13163

**Published:** 2019-02-20

**Authors:** Lukas Widmer, Elia Heule, Marco Colombo, Attila Rueegg, Adrian Indermaur, Fabrizia Ronco, Walter Salzburger

**Affiliations:** ^1^ Department of Environmental Sciences Zoological Institute University of Basel Basel Switzerland

**Keywords:** cichlid fish, community ecology, comparative analysis, diversity, lake tanganyika, monitoring, sampling, underwater visual census

## Abstract

1. Available underwater visual census (UVC) methods such as line transects or point count observations are widely used to obtain community data of underwater species assemblages, despite their known pit‐falls. As interest in the community structure of aquatic life is growing, there is need for more standardized and replicable methods for acquiring underwater census data.
2. Here, we propose a novel approach, Point‐Combination Transect (PCT), which makes use of automated image recording by small digital cameras to eliminate observer and identification biases associated with available UVC methods. We conducted a pilot study at Lake Tanganyika, demonstrating the applicability of PCT on a taxonomically and phenotypically highly diverse assemblage of fishes, the Tanganyikan cichlid species‐flock.
3. We conducted 17 PCTs consisting of five GoPro cameras each and identified 22,867 individual cichlids belonging to 61 species on the recorded images. These data were then used to evaluate our method and to compare it to traditional line transect studies conducted in close proximity to our study site at Lake Tanganyika.
4. We show that the analysis of the second hour of PCT image recordings (equivalent to 360 images per camera) leads to reliable estimates of the benthic cichlid community composition in Lake Tanganyika according to species accumulation curves, while minimizing the effect of disturbance of the fish through SCUBA divers. We further show that PCT is robust against observer biases and outperforms traditional line transect methods.

## Foreign Language Abstract Zusammenfassung

1. Trotz der bekannten Schwächen dieser Methoden, werden Unterwasser‐Zensusverfahren, wie zum Beispiel das Linien‐Transsekt oder die Punkzählung, feldübergreifend benutzt um Daten zu Lebensgemeinschaften und deren Artenzusammensetzung zu erheben. Mit zunehmendem Interesse an der Struktur von aquatischen Lebensgemeinschaften wächst auch die Nachfrage nach standardisierten und replizierbaren Methoden um solche Daten zu erheben.
2. Wir präsentieren hier eine neue Methode, das Punkt‐Kombinations Transsekt (PCT), welches auf Aufnahmen von kleinen Unterwasserkameras beruht und dadurch den Einfluss des Experimentators und Identifizierungsfehler minimiert. Zur Illustration dieser Methode haben wir am Tanganjika‐See in Sambia eine Pilotstudie durchgeführt um die Anwendbarkeit der PCT auf einer taxonomisch und phänotypisch diversen Lebensgemeinschaft von Fischen, den Buntbarschen, zu demonstrieren.
3. 17 PCTs wurden durchgeführt, jeweils bestehend aus fünf GoPro Kameras. Mit den Bildern konnten insgesamt 22,867 Buntbarschindividuen identifiziert und 61 Arten zugewiesen werden. Als Qualitätsanalyse haben wir die gesammelten Daten mit denen von klassischen Linien‐Transsekt Studien verglichen, welche in unmittelbarer Nähe des Standortes der Pilotstudie durchgeführt wurden.
4. Wir zeigen, dass durch die Analyse der jeweils zweiten Stunde, der im PCT aufgenommenen Bilder (entspricht 360 Bildern pro Kamera) eine solide Quantifizierung der benthischen Buntbarsch Lebensgemeinschaft im Tanganjika See möglich ist. Zudem wird der Störeffekt durch Taucher minimiert. Ausserdem können wir demonstrieren, dass PCT eine gewisse Resistenz gegen subjektive Datenverzerrung durch einen Beobachtereffekt hat und in dieser Hinsicht konventionelle Linien‐Transsekte überlegen ist.

## 1 INTRODUCTION

Underwater visual census (UVC) methods such as line transect (Brock, 1954) or point count observation (Samoilys & Carlos, 1992, 2000) are widely applied in ecology and, today, represent a standard approach for the non‐invasive assessment of underwater communities, particularly of fish. In order to obtain UVC data the observation is typically performed directly by SCUBA divers (or snorkelers), who record the presence and abundance of the species under investigation following standardized procedures (Colvocoresses & Acosta, 2007; Dickens, Goatley, Tanner, & Bellwood, 2011; Whitfield et al., 2014). A major drawback of UVC applications involving human observers is that these are subject to a number of biases, which are – depending on the strategy used – difficult or impossible to avoid. For example the presence of the observer can itself have a strong effect on the local fish community by altering fish behaviour (Dickens et al., 2011; Pais & Cabral, 2017). Observer swimming speed and distance to substratum have been reported as additional factors that can influence the observational results of transect studies (Edgar, Barrett, & Morton, 2004). Another potential problem is observer expertise and subjectivity, typically resulting in data skewing towards well‐known species (Thompson & Mapstone, 1997; Williams, Walsh, Tissot, & Hallacher, 2006). These problems can largely be overcome using digital imaging technologies that are observer‐independent and generate underwater images or video footage that can subsequently be analysed (Pereira, Leal, & de Araújo, 2016). Using digital information has the additional advantage that the raw data can be stored and re‐evaluated if desired, thus facilitating repeatability and reproducibility of the results.

The application of camera‐based census methods in the aquatic realm is, however, much more challenging than in terrestrial ecosystems. For example aquatic habitats are typically much less accessible, and light penetration and visibility are much lower in water than in air. Cameras for underwater use need to be specifically equipped and protected, which subsequently makes the handling, installation and recovery of cameras more difficult; standard procedures used in census surveys in terrestrial habitats cannot easily be applied underwater (e.g. the use of motion sensors would cause cameras to fire constantly due to water movement and/or suspended particles, whereas the use of artificial or flash light would bias the observations by attracting or scaring off certain individuals). Despite the general difficulties, several camera‐based census methods are available to date specifically tailored towards underwater use. The STAVIRO method introduced by Pelletier et al. (2012), for instance, consists of an encased camera revolving about itself on a motor, taking images of a circular area in accordance with the principles of point observations. Although bias by observer presence is reduced or entirely eliminated, the moving object of the STAVIRO apparatus might still alter fish behaviour (Mallet, Wantiez, Lemouellic, Vigliola, & Pelletier, 2014). The often‐used Baited‐Remote Underwater Video (BRUV) technique involves video surveillance of bait, which is placed in a particular habitat (Lowry, Folpp, Gregson, & Mckenzie, 2011; Unsworth, Peters, McCloskey, & Hinder, 2014). The resulting footage is then used to estimate fish abundance. Although under certain circumstances this might be a valuable approach, it is not suitable for observing a community as a whole, as there is a species‐specific bias through the bait used (Wraith, Lynch, Minchinton, Broad, & Davis, 2013).

**Figure 1:**
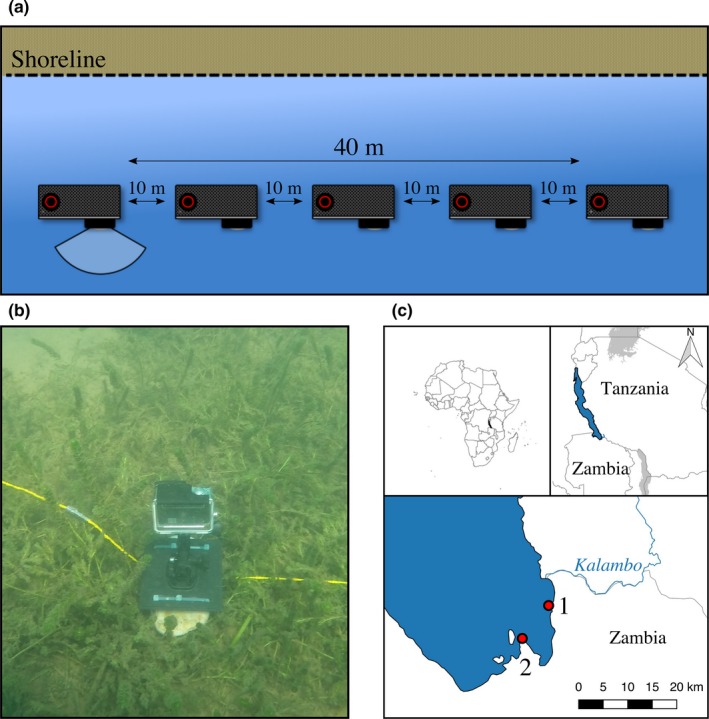
(a) Design and set up of a Point‐Combination Transect (PCT) as used in the pilot. GoPros face perpendicular away from shoreline. The focal angle of 120° is illustrated for one camera. (b) Underwater image of GoPro placement. (c) Map of sampling locations at Lake Tanganyika in Zambia, Africa: 1 – This study, MetA, and MetC; 2 – MetB (See section 2.4.3 for corresponding comparitive studies)

Here we introduce a novel approach, the Point‐Combination Transect (PCT) method (Figure 1a,b), which incorporates elements of conventional UVC line and point transects with digital underwater imaging tools. We demonstrate the wide applicability of PCT by employing it on a rather complex assemblage of fishes, the species flock of cichlid fishes from Lake Tanganyika in East Africa. This fish community is dominated by species that strongly interact with the substrate, exemplified through numerous substrate breeders or algae scrappers; but even highly mobile and pelagic species interact closely with the benthos, for example when predating others or during spawning (Konings, 1998). Our novel approach is based on small, automated digital cameras in underwater housings that are placed on the benthos and aligned along a given distance at a set depth level. The PCT method enables a researcher to observe several spatially close communities simultaneously by automatically recording images in a defined time lapse. Once the cameras are placed, there is no further disturbance by SCUBA divers and no interaction of the camera with its surroundings, including no movement and no visual or audible signalling. We show how with relatively little monetary and timely investment, valuable and robust data on fish community structures can be collected, even at remote places and under demanding field conditions.

## 2 MATERIALS AND METHODS

### 2.1 Study site

The pilot was conducted at Lake Tanganyika, East Africa. The study site was restricted to the bay off Kalambo Falls Lodge located close to the mouth of Kalambo River (8°37′36″ S, 31°12′2″ E) in northern Zambia (Figure 1c). This bay was chosen for its diversity in habitats present within close proximity and its accessibility from Kalambo Falls Lodge. Furthermore, the bay is subjected to moderate fishing pressure only, primarily targeting non‐cichlid fish species. Hence we assumed to observe a relatively undisturbed, local fish community bereft of extensive anthropogenic influences. The study area comprises a diverse set of environments, such as predominantly rock‐ or sand‐covered habitats; areas with an intermediate coverage of the lakebed; or vegetation dominated habitats. PCTs were conducted on a variety of depth levels, ranging from <1 m up to 21 m.

### 2.2 Point‐Combination Transect settings

The technical equipment for our PCT consisted of GoPro cameras (Hero 3+ Silver Edition, Hero 4+ Silver Edition, © GoPro, Inc.), each equipped with a 16 GB microSD card (ScanDisk) ensuring sufficient storage capacity for high‐quality image storage. The protective housing provided by the supplier is waterproof to a depth of 40 m, making additional underwater housing unnecessary. The cameras were mounted in their housing on the supplied stand and fixed to a small rock (approximate dimensions: length = 15 cm, width = 15 cm, height = 5 cm) to provide negative buoyancy, immobility and stability once placed underwater on the lakebed (Figure 1b).

The setup for a PCT consists of five GoPro cameras positioned in a distance of 10 m of each other along a marked cord (total length of the transect: 40 m) (Figure 1a). The length of 40 m for one transect was chosen to ensure safe placement of two PCTs within the bottom time restrictions for a diver pair as advised by PADI. The deployment of a complete PCT was feasible within 10 to 15 min not considering the time to reach the starting point of the PCT and the return dive. The study area of the pilot was initially classified into major substrate types. Based on these classifications, the SCUBA pairs dove into a substrate type to target a certain depth and started the PCT at a random point. As depth was the main criteria for the starting point within a substrate type, distance between PCTs was directly linked to the slope of the lakebed. The cameras were placed perpendicular to the shoreline facing the open water (or facing the shoreline if depth of camera was 1 m or less; Figure 1a) and immediately turned on after setting up. The exact depth of each camera was determined with a diving computer and recorded on a dive slate.

The cameras were left for roughly three hours at their observation point and images were automatically recorded every 10 s during this entire period. The image recording was set to maximum quality, that is, 4,000 × 3,000 pixels for the GoPro Hero 4+ model and 3,680 × 2,760 pixels for the GoPro Hero 3+. No flash was used and all visual and acoustic signals of the cameras were suppressed to prevent attraction or repulsion of fish. The observational area of one camera was considered a segment of a circle and therefore could be estimated using the radius *r* and focal angle Θ of the lens. The radius was approximated to 3.0 m (due to visibility limitations and variations among cameras), resulting into an observational area of 5.5 m^2^ (based on the focal angle of GoPro cameras of 120°). The deployment of a signalling buoy 2 m from the end of the transect line ensured the secure retrieval of transects. Images were subsequently copied to two separate 1 TB hard drives for storage and backup. Within the framework of the pilot a total of 17 PCTs were conducted during July and August in two consecutive years (2014 and 2015).

### 2.3 Image analysis

Prior to any analysis, an image selection based on the last appearance of SCUBA divers on the images was performed to minimize any influence on the local fish assemblage that may have been caused by human presence. Whenever feasible the first 60 min of the recordings were discarded to guarantee observation of an undisturbed community and the second 60 min (360 images) were extracted for visual inspection. Due to shorter battery runtimes or other technical issues, this criterion could not be met for all cameras. In cases where cameras recorded images for less than 120 min, we extracted a frame of 360 images maximizing time to last appearance of a SCUBA diver (Table S1). The selected set of 360 images per camera was transferred onto a server, whereby each image received a unique ID consisting of PCT‐, GoPro‐, and image number (e.g. 005‐21‐00130023). The images were processed in a custom‐made web platform, linked to a SQL database to provide safe and efficient storage. All 360 images per camera were individually analysed, whereby cichlid specimens were identified to species level and counted according to a set of predefined criteria (Table 1). Both, adults and juveniles were included in the analysis. In cases where species identification was not reliably possible, the respective specimen was classified into the next higher taxonomic rank (genus or tribe). Our custom‐made analysis tool also included a “review” button to highlight questionable specimens for later inspection by a taxonomy expert artificial intelligence.

**Table 1:** Compilation of criteria for specimen selection and identification during image analysis

Species identification and count	
Individual is **IDENTIFIED** and **COUNTED** if the fish is: fully visible (entire body, head to caudal fin) facing squarely (body ~ 90°–135°) to camera a cichlid^a^ neither omitted/marked as unidentifiable (see criteria below)	
**OMITTED** completely if: partially on picture or partially covered by stone or other structures (e.g. vegetation) body angles more than ~135° from camera	marked as **UNIDENTIFIABLE** if: body angles less than 135° from camera clearly a cichlid passed criteria for omission but contortion or velocity impedes on identification

Note

a Non‐Cichlids were selected under the same criteria, no identification was done however.

For habitat characterization individual images from each PCT were overlaid with a 10 × 10 rectangular grid‐layer implemented in the web interface. Habitat parameters were visually characterized by first categorizing each rectangle into visible structure (e.g. lakebed, rock formations) or open water. The visible structure was then examined for rock, sand, and vegetation coverage. Every rectangle was assigned a single category corresponding to the most dominant feature within. Topological features such as rock size and frequency were also quantified (Table S2).

### 2.4 Data analysis

#### 2.4.1 Data preparation

Following image analysis, the fish abundance was summarized for every camera. A notorious problem for point observation data is the overestimation of population sizes due to multiple counting of the same individuals (Ward‐Paige, Flemming, & Lotze, 2010). To reduce the effect of multiple counting, the maximum number of individuals (MaxN; Merrett, Bagley, Smith, & Creasey, 1994; Wartenberg & Booth, 2015) per species on a single image out of the 360 images was taken as the species count for the given camera. As a comparative measure we calculated the mean per species over 360 images, using only non‐zero values. We subjected data of each camera to additional scrutiny by filtering for species that occurred only on three or less images and verified these findings through a second visual inspection of the images in question.

#### 2.4.2 Method evaluation

To evaluate the robustness of the PCT method, we first computed a species accumulation curve (SAC) in R (R Development Core Team, 2016) using the *specaccum* function from the vegan package version 2.4‐5 (Oksanen et al., 2018) (10,000 permutations) for each camera. The resulting curves were fitted to a quadratic response plateau model using *nlsfit* implemented in the easynls package version 5.0 (Arnhold, 2017) to evaluate if and after which number of images species richness R reaches a plateau for each of the SACs. The computed SAC data were additionally used to predict species richness for an increased sampling effort of 720 images (two hours of analysis) and to illustrate the theoretical gain in species. The same procedure was applied to the number of cameras within a PCT, for up to 20 cameras. The issue of a possible observer bias was also investigated: First, a comparison of observed species was performed to detect discrepancies in identified species between two observers (LW, EH). Second, an ANOVA was performed to test the difference between the two observers in the raw fish count and species richness data. Finally, we examined possible differences in fish count and species richness data between the first and second hour of recording by comparing 1,000 random sets of 12 images from the first and second hour of recordings, using ANOVAs.

#### 2.4.3 Comparison to previous studies

In order to assess the power of PCT, we compared the results of our pilot experiment to three traditionally performed transect studies conducted in the close vicinity to our study site (Janzen et al., 2017; Sturmbauer et al., 2008; Takeuchi, Ochi, Kohda, Sinyinza, & Hori, 2010). Hereafter, we will refer to these studies as follows; MetA – Sturmbauer et al. (2008), MetB – Takeuchi et al. (2010), MetC – Janzen et al. (2017). MetA and MetC were completed within a 500 m distance from our study site, whereas MetB monitored an area of 400 m^2^ for over 20 years at Kasenga Point (8°43′ S, 31°08′ E), which is located roughly 15 km from our location (see Figure 1c). Due to their close proximity and general setup, these studies seem well suited to evaluate the efficiency of our PCT methodology. All three studies used conventional UVC SCUBA diver line transects as a means to observe and quantify the fish population and species diversity at their respective location. To maximize comparability among the studies, we only considered data from a depth level between 1 to 5 m and rocky habitat (rock coverage >75%) (Table S2). Species richness and the Shannon diversity index were calculated for all studies using the *diversity* function in vegan. Variances in observed fish density among the three studies were compared using a Mann–Whitney *U* test on the count data per species. MetA provided no actual counts for species observed three times or fewer, hence we assumed a value of three for these species in the above‐mentioned analyses. As an additional evaluation of the appropriateness of the PCT approach, we tested for a size‐dependent observation bias. To this end, we categorized the observed species into two size classes based on their standard length (SL). The mean SL of at least 10 specimens per species, extracted from the Tanganyika cichlid collection at the Zoological Institute of the University of Basel, was used for this comparison.

## 3 RESULT

### 3.1 Pilot study

The 17 PCTs of this study yielded data from 78 cameras, that is, 28,080 images for the subsequent analysis of the cichlid community at the study site (Exemplary images: Figure S3). The PCTs encompass depths from 1 m to 21 m and three major habitat types: sandy, rocky and intermediate. 17,322 individual fish were identified to species level, 1,566 to genus level and 5,269 fish could not be identified on the images. The MaxN statistics of the raw count data resulted in 3,030 specimens at the species level (2,761 specimens if using the mean), 124 at the genus level and 324 at the tribe level. In total 61 cichlid species were recorded in the 2 years of this pilot on three different habitat types.

### 3.2 Method evaluation

**Figure 2:**
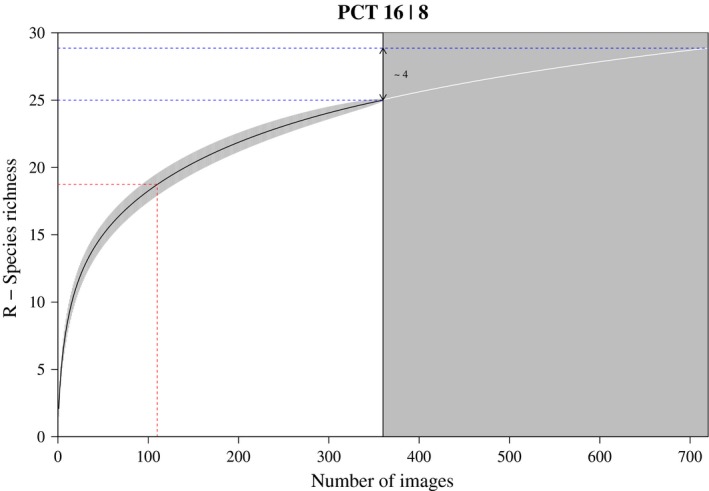
Exemplary SAC plot of PCT No 16 and camera No 8. On white background the computed SAC, with indication of reaching 75% of the total species number observed (red dashed line). On grey background the predicted gain in species number, calculated using the Weibull growth model. The theoretical gain in species richness is indicated between the two blue lines (blue dashed line)

The species accumulation curves (SACs) were calculated for 64 cameras (14 cameras were excluded from the analysis due to the small number of species recorded) (Table S4). The SACs of 53 cameras reached the plateau of species richness saturation before 360 images. The resulting image number for saturation was between 107 and 360 with an average of 262 ± 75 images. The remaining 11 SACs would reach the plateau between 362 and 409 images, with an average of 380 ± 16 images. A threshold of 75% of species observation was achieved after 128 ± 50 images for all 64 cameras (Figure 2). The theoretical gain of increased sampling effort in species richness could be computed for 50 cameras and ranged from 0.00 to 4.64 (± 1.19). For the SACs of the PCTs, none displayed a plateau, but on average 75% of species were observed after half of the cameras were analysed (Table S4). Boosting the camera number to 20 per PCT predicted a gain in species richness between 2.04 and 10.03 (± 2.62).

The comparison of 1,000 subsets of 12 images each from the first and second hour of recordings provided no evidence for any significant effect of elevated disturbance in the first hour after installation (Table S5).

**Figure 3:**
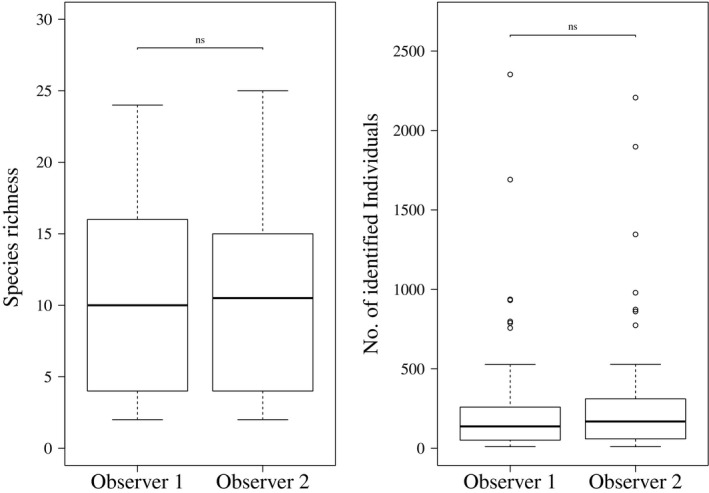
Boxplots of the comparison between two independent observers (Observer 1: L.W., Observer 2: E.H.) of 17 PCTs (78 cameras). Comparison of species richness R: ANOVA *p* = 0.68. Total of identified cichlid fish: ANOVA *p* = 0.72

The difference in the number of observed species between the two independent observers was non‐significant (ANOVA, *F* = 0.18, *p* = 0.68), as was the difference in actual fish counts (ANOVA, *F* = 0.13, *p* = 0.72) (Figure 3). Among the 61 taxonomically assigned species only two differences were registered between the two observers.

### 3.3 Comparison to previous studies

**Figure 4:**
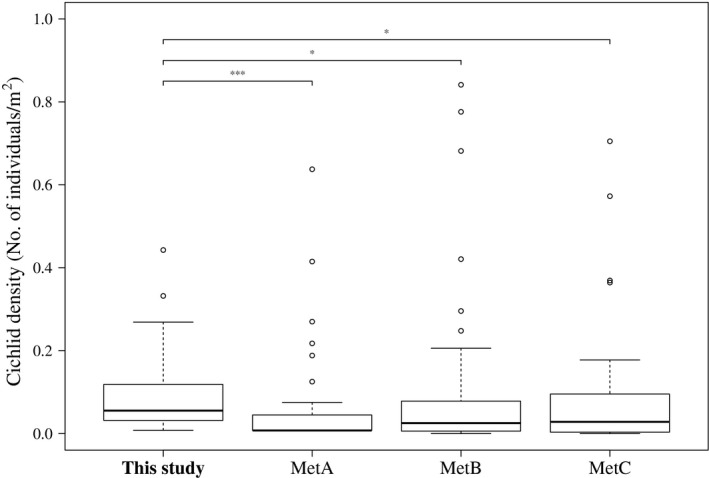
Boxplot of comparison among cichlid density for the pilot and the three comparative studies. Densities calculated for each species based on count data and area of observation: This study (125 m^2^), MetA (400 m^2^), MetB (180 m^2^), MetC (1,200 m^2^) (*^**^ *p* = 0.00, *p < 0.05)

Of the 17 PCTs used in this study, five PCTs (8,280 images) were considered for the comparison to previous studies due to the similar depth range (up to 5 m) and habitat structure (rock coverage higher than 75%) (Table S2). Although the five PCTs analysed here covered a much smaller area, we detected more species than MetA or MetC; only in the 20‐years census of MetB more species were found (Table 2). The observed density for cichlids was significantly higher in the present study compared to the three studies based on conventional UVC methods (MetA, Mann–Whitney *U* test, *W* = 1,347, *p* = 0.00; MetB, Mann–Whitney *U* test, *W* = 1,483, *p* = 0.02; MetC, Mann–Whitney *U* test, *W* = 994, *p* = 0.03) (Figure 4). If considering only species for which four or more individuals were observed, as executed in MetA, species richness is highest with PCT (Table 3). The observed cichlid densities, however, were then only significantly higher compared to MetA (Figure S6). Finally, no significant size bias through more frequent observation of smaller species was observed for PCT (Mann–Whitney *U* test, *W* = 186, *p* = 0.44) (Figure S7).

**Table 2:** List of species observed in different studies as count and density data. Density calculations were based on 126.5 m^2^ for this study, 400 m^2^ for MetA, 180 m^2^ for MetB, and 1,200 m^2^ for MetC (*: species not occurring at the study location, +: for MetA a count of 3 or less individuals from a particular species)

	This study		MetA		MetB		MetC	
	Count	Density	Count	Density	Count	Density	Count	Density
*Altolamprologus compressiceps*	9	0.071	5	0.013	2.0	0.005	22	0.018
*Aulonocranus dewindti*	4	0.032	166	0.415	53.2	0.133	4	0.003
*Boulengerochromis microlepis*			+					
*Callochromis macrops*	6	0.047	+				3	0.003
*Chalinochromis brichardi*	17	0.134	19.5	0.049			86	0.072
*Ctenochromis horei*	6	0.047	+		1.5	0.004	15	0.013
*Cunningtonia longiventralis*	2	0.016			0.6	0.002		
*Cyathopharyxn foae*	2	0.016						
*Cyathopharyxn furcifer*	3	0.024	75.3	0.188				
*Cyprichromis zonatus*								
*Cyprichromis coloratus*								
*Cyprichromis leptosoma*	14	0.111			5.1	0.013	1	0.001
*Eretmodus cyanostictus*	18	0.142	50.1	0.125	122.7	0.307	443	0.369
*Gnathochromis pfefferi*	12	0.095	+		12.2	0.031		
*Haplotaxodon microlepis*	3	0.024	+		4.6	0.012	2	0.002
*Interchromis loocki*	5	0.040			14.1	0.035		
*Julidochromis marlieri*			+					
*Julidochromis ornatus*	4	0.032	10	0.025	11.7	0.029	97	0.081
*Lamprologus callipterus*	3	0.024	+		8.1	0.020	22	0.018
*Lamprologus lemairii*	2	0.016	+		4.4	0.011		
*Lepidiolamprologus attenuatus*			+		5.0	0.013	4	0.003
*Lepidiolamprologus cunningtoni*					0.2	0.001		
*Lepidiolamprologus elongatus*	14	0.111	8	0.020	14.1	0.035	6	0.005
*Lepidiolamprologus kendalli*								
*Lepidiolamprologus mimicus*								
*Lepidiolamprologus profunidcula*					0.7	0.002		
*Limnotilapia dardennii*	10	0.079	+		12.8	0.032		
*Lobochilotes labiatus*	20	0.158	22	0.055	18.5	0.046	50	0.042
*Neolamprologus fasciatus*	29	0.229	+		75.7	0.189	131	0.109
*Neolamprologus furcifer*			+		1.0	0.003		
Neolamprologus buescheri*								
*Neolamprologus caudopunctatus*			+		10.8	0.027	33	0.028
*Neolamprologus cylindricus*			+		1.9	0.005	4	0.003
*Neolamprologus modestus*	3	0.024	3.6	0.009			47	0.039
Neolamprologus mustax*					1.3	0.003		
*Neolamprologus obscurus*					0.2	0.000		
Neolamprologus petricola*					1.4	0.004		
*Neolamprologus prochilus*					0.6	0.001	2	0.002
*Neolamprologus pulcher*	6	0.047			4.3	0.011	123	0.103
*Neolamprologus savoryi*	8	0.063			1.1	0.003	34	0.028
*Neolamprologus sexfasciatus*					4.5	0.011		
*Neolamprologus tetracanthus*	5	0.040	16.5	0.041	0.2	0.001	122	0.102
*Opthalmotilapia nasuta*	4	0.032	+					
*Opthalmotilapia ventralis*	8	0.063	87	0.218	196.8	0.492		
*Oreochromis tanganicae*			+					
*Paracyprichromis brieni*					3.4	0.008		
*Perissodus microlepis*	21	0.166	+		31.4	0.079		
*Petrochromis ephippium*	7	0.055					41	0.034
*Petrochromis famula*	4	0.032			4.1	0.010	19	0.016
*Petrochromis fasciolatus*	8	0.063			12.9	0.032	45	0.038
*Petrochromis polyodon*	10	0.079			23.3	0.058	19	0.016
Petrochromis trewavasae*					34.1	0.085		
*Plecodus straeleni*			+		2.7	0.007		
*Pseudosimochromis curvifrons*	16	0.126	+		4.6	0.012		
*Simochromis diagramma*	28	0.221			44.6	0.112	54	0.045
*Telmatochromis temporalis*	15	0.119	+		12.1	0.030	846	0.705
*Telmatochromis vittatus*	15	0.119	+		139.7	0.349	160	0.133
*Tropheus moorii*	56	0.443	108	0.270	151.4	0.379	437	0.364
*Tylochromis polylepis*			+					
*Variabilichromis moorii*	42	0.332	255	0.638	367.7	0.919	687	0.573
*Xenotilapia boulengeri*	5	0.040	+				107	0.089
Xenotilapia papilio*					3.1	0.008		
*Xenotilapia spilopterus*	7	0.055	+		37.0	0.093	213	0.178
Total	451		826		1,463.4		3,879	

**Table 3:** Summary table of this study and three studies used for comparison. Area of observation (AoO), species richness (R), species richness for species with 4 or more individuals sighted (R^4^) and Shannon‐Diversity Index (SI) are shown. For MetB species richness (R) except species that do not occur at location of this study is shown in brackets

Study	AoO	R	R^4^	SI
This study	125 m^2^	39	32	3.30
Sturmbauer et al. (2008)—MetA	400 m^2^	37	12	2.37
Takeuchi et al. (2010)—MetB	180 m^2^	46 (41)	30 (29)	2.65
Janzen et al. (2017)—MetC	1,200 m^2^	32	28	2.56

## 4 DISCUSSION

In this study, we present a novel method – PCT – specifically tailored towards the examination of underwater communities, particularly fish. Interest in the community structures of aquatic species assemblages is increasing and is no longer restricted to ecology but gains importance in other fields such as evolutionary and conservation biology (Pillar & Duarte, 2010; Schmidt, White, & Denef, 2016; Yang, Powell, Zhang, & Du, 2012; Yunoki & Velasco, 2016). This increased interest calls for appropriate, standardized, and replicable methodologies to acquire such data.

Our new method involves small, easily available digital cameras (GoPro) that are set in the benthic environment of a water body and record images in a set time‐interval to capture the local fish community. Two SCUBA divers set out five cameras along a line of 40 m, record the depth of each camera, and then leave the water to ensure minimal disturbance during observation time. We verified our new method PCT in a pilot study, covering two consecutive field seasons (2014 and 2015), in which we aimed to quantify the cichlid fish community of Lake Tanganyika at Kalambo Falls Lodge. Furthermore we compared the results to studies using conventional UVC line transect approaches, which were conducted in close proximity to our own study site.

In the 17 PCTs performed, a total of 22,867 cichlid fish were identified, of which 17,322 (75.8%) could be assigned to species level (6.8% to genus and 17.4% to the next higher taxonomic rank). In our pilot, we analysed 360 images per camera, a number that appears to be sufficient to capture most of the species present, considering the results from our SAC analysis. For the majority of the cameras we found that reducing the number of analysed images by a 100 would not have impacted the species composition compared with the total of 360 images (Table S4). However, the sampling effort of 360 images seems a good compromise between establishing a robust dataset and the time‐consuming image analysis. As a measure to reduce the effect of multiple counting of individuals we used MaxN for each species. This approach is arguably prudent, however, we aimed to illustrate that even conservatively analysed, PCTs are able to outperform conventional methods. MaxN is favoured, as a comparison with the species mean per camera suggests an underestimation of the specimen count by the mean metric (Figure S8). Regarding the number of cameras used within a PCT, an increase would most certainly lead to an increase in observed species richness R as suggested by the SACs of the PCTs. However, extending a PCT in such a manner would not be feasible for all depth levels due to bottom time restrictions and diver safety.

A main advantage of our PCT methodology is the exclusion of different observer‐based biases. Our method allows the omission of the first hour of recordings, or rather the maximization of time between beginning of analysis and “last seen diver” (an element added to our approach purposefully to reduce bias introduced by human presence). As we did not find any differences in the species composition for the omitted images and the data used for the analysis, however, it appears to be an excessive restraint. Observer expertise has been discussed in various studies and shown to directly influence count data and identification efforts (Thompson & Mapstone, 1997; Williams et al., 2006). In the case of PCT, the difference between two independent observers proved to be insignificant (Figure 3). In 28,080 images and 61 cichlid species only two individuals were assigned to different species by the two observers. Even count data were the same between the two observers, likely as a result of the highly standardized approach to identify and count the cichlid fishes on the images.

To compare directly with studies done in a similar location we stripped down our data to only five PCTs, which reduced the number of species observed in the full pilot (61 to 39 species, see Section 3.1). In terms of species richness, PCT outperformed the conventional UVC line‐transect for both studies done in very close proximity to our study location and is virtually tantamount to the 20‐year census done by MetB. This result clearly indicates the power of the PCT in comparison with the conventional UVC line‐transect methodology. Taking into account the difference in the area covered with UVC line‐transect and PCT this impression is further strengthened: Even though our PCTs covered only a fraction of the area of observation compared to the three comparative studies, they captured as many species as the average of the 20 year‐census of MetB and more than double the species of MetA, suggesting that traditional UVC line transect approaches fail to record all species present at study site. The lack of specifications in the comparative studies and the different nature of observations – continuous observation in traditional transects of approximately 12 min (Samoilys & Carlos, 1992) vs. 360 snapshots taken during 60 min (PCT) – made it unreliable to directly compare sampling effort as a function of time of observation. Although time surely must have an effect, we believe that the distinct feature of PCT, the absence of divers during recording, surpasses that effect in regard to the observed species richness. Regarding count data, all comparative studies reported markedly greater numbers. While count data were higher, we would like to stress that they were mainly driven by a few species, such as the shoaling females of ectodini genera *Cyathopharynx* and *Ophthalmotilpia* or densely occurring *Variabilichromis moorii*; it has previously been shown by Pais and Cabral (2017) that abundance of schooling or in this case shoaling fish is usually overestimated in traditional census methods. After taking into account the area covered in the studies, we compared fish densities and again found that PCT outperformed the conventional methods by a fair margin. Furthermore, we believe that even though a GoPro camera only covers a fraction of the area usually covered by conventional UVC dives, we are able to capture the fish community structure in gross detail and in a mostly undisturbed state. As mentioned above, PCT delivers accurate local abundance data of the species community. Using abundance and standard length (SL), biomass may be approximated, although prior information on SL is necessary as no length measurements can be taken from non‐stereo images (as performed on stereo images by Wilson, Graham, Holmes, MacNeil, & Ryan, 2018). An alternative approach could be the measuring of landmarks while setting up PCT to allow researchers to measure individuals *a posteriori*. As this was not the aim of this study, we are unable to provide more detailed information here.

Looking in depth at the species that were observed, we investigated if camera position biased our data to small and benthic species. We did, however, not find any evidence that would support this. When comparing this aspect directly with the other studies and the UVC strip transect, there was no evidence for a significant shift towards small species. The general set up of the PCT does suggest a focus on benthic communities; however, our method is able to capture mobile and pelagic species as well (Figure S9). We thus see the advantage of the observational success not depending on the size or position in the water column of the fish, as illustrated within this study. However, it is advisable to select target species with a certain degree of dependence on the substrate.

To date, several approaches exist to incorporate the use of electronic equipment and therefore reduce a number of biases associated with conventional UVC used for ecological observation of underwater communities. For example TOWed Video (TOWV) is used to monitor communities by recording footage as the cameras are pulled through the habitat (Mallet & Pelletier, 2014). However, regarding observer presence, the use of cameras would not have markedly benefited the quality of the collected data in this instance, as firstly, depending on the depth, heavy surface disturbance has to be considered, and more importantly the moving, baited object pulled through the fish community might selectively attract some fish species over others (Pais & Cabral, 2017; Pereira et al., 2016). Therefore, abundance and species richness data of the habitat in question might not reflect reality. A different approach was introduced in 2012 (STAVIRO; Pelletier et al., 2012) using stationary cameras that rotate to simulate a point transect, presumably eliminating the bias of observer presence. This approach marginally failed to show its superiority to general UVC techniques and might still contain bias through its moving apparatus (Mallet et al., 2014). In contrast, an indication for the inconspicuousness of our outlined methodology (PCT) is that a number of species difficult to monitor could be captured on camera, for example pelagic predators such as *Bathybates fasciatus* and the African tigerfish *Hydrocynus vittatus*, the latter of which was never directly observed in this area (personal observation) in 10 years diving at this location, or the shy cichlid species *Neolamprologus prochilus* that usually remains under rocks and is therefore rarely seen (Konings, 1998).

Considering all approaches using cameras, including PCT, it is important to note that the recording of the underwater image material is the smaller part of data collection, followed by a time intensive period of images analysis. The main advantages of PCT compared to other camera‐based approaches are its compact design, its cost effectiveness, its standardized setup and handling, as well as its ability to deliver robust digital data, making PCT well suited for the observation of underwater communities even under difficult field conditions.

## AUTHORS’ CONTRIBUTIONS

L.W., E.H., M.C. and W.S. conceived and supervised the study, all co‐authors conducted the fieldwork, L.W. constructed the image analysis tool and SQL database, L.W. and E.H. processed the images, with A.I. reviewing difficult cases. F.R. provided data on standard length, L.W. analysed the data, and wrote the manuscript with feedback from all co‐authors.

## DATA ACCCESSIBILTY

All raw count data used in this study including a separate species list are available from the Dryad Digital Repository https://doi.org/10.5061/dryad.1kr7759 (Widmer et al., 2019).
