# Supplementary material for: Point‐Combination Transect (PCT): Incorporation of small underwater cameras to study fish communities
Source: Methods Ecol Evol. 2019 Feb 20;10(6):891–901. doi: 10.1111/2041-210X.13163 (PMC6582616; doi:10.1111/2041-210X.13163)
Supplement: Supplementary file 1 [file MEE3-10-891-s001.docx]

Table S1: List of placed cameras for each PCT, including start, end, runtime, start of analysis (S_A) and end of analysis (E_A). maxTime indicates where the time to last seen diver was maximised.

| **PCT** | **GoPro No** | **Start**  **(hh:mm)** | **End**  **(hh:mm)** | **Runtime**  **(hh:mm)** | **S_A**  **(hh:mm)** | **E_A**  **(hh:mm)** | **maxTime** |
| --- | --- | --- | --- | --- | --- | --- | --- |
| 6 | 1 | 10:32 | 14:39 | 4:07 | 11:33 | 12:32 |  |
| 6 | 2 | 10:40 | 14:49 | 4:09 | 11:42 | 12:41 |  |
| 6 | 3 | 10:44 | 14:53 | 4:09 | 11:46 | 12:45 |  |
| 6 | 4 | 10:46 | 14:52 | 4:06 | 11:48 | 12:47 |  |
| 7 | 1 | 08:07 | 10:57 | 2:50 | 09:09 | 10:08 |  |
| 7 | 2 | 08:10 | 10:56 | 2:46 | 09:12 | 10:11 |  |
| 7 | 3 | 08:11 | 12:04 | 3:53 | 09:13 | 10:12 |  |
| 7 | 4 | 08:16 | 09:49 | 1:33 | 08:28 | 09:27 | ✔ |
| 7 | 5 | 08:18 | 11:23 | 3:05 | 09:20 | 10:19 |  |
| 8 | 1 | 10:14 | 12:23 | 2:09 | 10:42 | 11:41 | ✔ |
| 8 | 2 | 10:18 | 12:24 | 2:06 | 10:40 | 11:39 | ✔ |
| 8 | 3 | 10:20 | 12:24 | 2:04 | 10:39 | 11:38 | ✔ |
| 8 | 4 | 10:27 | 12:26 | 1:59 | 10:48 | 11:47 | ✔ |
| 10 | 6 | 16:15 | 17:43 | 1:28 | 16:43 | 17:42 | ✔ |
| 10 | 7 | 16:22 | 17:44 | 1:22 | 16:44 | 17:43 | ✔ |
| 10 | 8 | 16:25 | 17:45 | 1:20 | 16:45 | 17:44 | ✔ |
| 10 | 9 | 16:27 | 17:46 | 1:19 | 16:46 | 17:45 | ✔ |
| 10 | 10 | 16:28 | 17:46 | 1:18 | 16:47 | 17:46 | ✔ |
| 11 | 11 | 11:10 | 12:53 | 1:43 | 11:52 | 12:51 | ✔ |
| 11 | 12 | 11:13 | 15:41 | 4:28 | 12:13 | 13:12 |  |
| 11 | 13 | 11:18 | 15:58 | 4:40 | 12:19 | 13:18 |  |
| 11 | 15 | 11:24 | 16:12 | 4:48 | 12:25 | 13:24 |  |
| 12 | 6 | 11:30 | 14:37 | 3:07 | 12:31 | 13:30 |  |
| 12 | 7 | 11:38 | 16:22 | 4:44 | 12:40 | 13:39 |  |
| 12 | 8 | 11:42 | 14:47 | 3:05 | 12:43 | 13:42 |  |
| 12 | 9 | 11:45 | 15:02 | 3:17 | 12:45 | 13:44 |  |
| 12 | 10 | 11:49 | 15:15 | 3:26 | 12:50 | 13:49 |  |
| 13 | 16 | 15:43 | 17:05 | 1:22 | 16:05 | 17:04 | ✔ |
| 13 | 17 | 15:50 | 17:06 | 1:16 | 16:07 | 17:06 | ✔ |
| 13 | 19 | 15:53 | 17:06 | 1:13 | 16:06 | 17:05 | ✔ |
| 13 | 20 | 15:54 | 17:05 | 1:11 | 16:05 | 17:04 | ✔ |
| 14 | 11 | 10:11 | 14:55 | 4:44 | 11:13 | 12:12 |  |
| 14 | 13 | 10:20 | 15:10 | 4:50 | 11:22 | 12:21 |  |
| 14 | 14 | 10:21 | 15:12 | 4:51 | 11:23 | 12:22 |  |
| 14 | 15 | 10:23 | 11:57 | 1:34 | 10:57 | 11:56 | ✔ |
| 15 | 6 | 10:31 | 13:08 | 2:37 | 11:32 | 12:31 |  |
| 15 | 7 | 10:35 | 12:17 | 1:42 | 11:17 | 12:16 | ✔ |
| 15 | 8 | 10:38 | 14:51 | 4:13 | 11:39 | 12:38 |  |
| 15 | 9 | 10:40 | 12:31 | 1:51 | 11:31 | 12:30 | ✔ |
| 15 | 10 | 10:42 | 14:48 | 4:06 | 11:43 | 12:42 |  |
| 16 | 6 | 10:25 | 12:26 | 2:01 | 10:35 | 11:34 | ✔ |
| 16 | 7 | 10:29 | 12:25 | 1:56 | 10:39 | 11:38 | ✔ |
| 16 | 8 | 10:32 | 12:24 | 1:52 | 10:42 | 11:41 | ✔ |
| 16 | 9 | 10:35 | 12:22 | 1:47 | 10:45 | 11:44 | ✔ |
| 16 | 10 | 10:38 | 12:17 | 1:39 | 10:48 | 11:47 | ✔ |
| 17 | 11 | 15:51 | 17:31 | 1:40 | 16:30 | 17:29 | ✔ |
| 17 | 12 | 15:50 | 17:30 | 1:40 | 16:29 | 17:28 | ✔ |
| 17 | 13 | 15:53 | 17:30 | 1:37 | 16:28 | 17:27 | ✔ |
| 17 | 14 | 15:53 | 17:28 | 1:35 | 16:26 | 17:25 | ✔ |
| 17 | 15 | 15:55 | 17:27 | 1:32 | 16:26 | 17:25 | ✔ |
| 18 | 11 | 10:48 | 12:31 | 1:43 | 10:58 | 11:57 | ✔ |
| 18 | 12 | 10:50 | 12:32 | 1:42 | 11:00 | 11:59 | ✔ |
| 18 | 13 | 10:54 | 12:35 | 1:41 | 11:04 | 12:03 | ✔ |
| 18 | 14 | 10:54 | 12:31 | 1:37 | 11:04 | 12:03 | ✔ |
| 18 | 15 | 10:55 | 12:32 | 1:37 | 11:04 | 12:03 | ✔ |
| 19 | 6 | 10:32 | 15:20 | 4:48 | 11:33 | 12:32 |  |
| 19 | 7 | 10:38 | 15:24 | 4:46 | 11:39 | 12:38 |  |
| 19 | 8 | 10:41 | 15:21 | 4:40 | 11:42 | 12:41 |  |
| 19 | 9 | 10:42 | 15:25 | 4:43 | 11:43 | 12:42 |  |
| 19 | 10 | 10:44 | 15:25 | 4:41 | 11:45 | 12:44 |  |
| 20 | 12 | 12:05 | 15:13 | 3:08 | 13:06 | 14:05 |  |
| 20 | 13 | 12:09 | 15:19 | 3:10 | 13:10 | 14:09 |  |
| 20 | 14 | 12:12 | 15:15 | 3:03 | 13:13 | 14:12 |  |
| 20 | 15 | 12:15 | 15:15 | 3:00 | 13:16 | 14:15 |  |
| 21 | 6 | 11:55 | 16:04 | 4:09 | 12:56 | 13:55 |  |
| 21 | 7 | 11:58 | 16:19 | 4:21 | 12:59 | 13:58 |  |
| 21 | 8 | 12:02 | 15:48 | 3:46 | 13:03 | 14:02 |  |
| 21 | 9 | 12:04 | 15:46 | 3:42 | 13:05 | 14:04 |  |
| 21 | 10 | 12:06 | 15:44 | 3:38 | 13:07 | 14:06 |  |
| 22 | 16 | 10:54 | 15:26 | 4:32 | 11:04 | 12:03 |  |
| 22 | 17 | 10:58 | 15:26 | 4:28 | 11:08 | 12:07 |  |
| 22 | 18 | 11:00 | 15:25 | 4:25 | 11:10 | 12:09 |  |
| 22 | 19 | 11:02 | 15:23 | 4:21 | 11:12 | 12:11 |  |
| 22 | 20 | 11:05 | 15:20 | 4:15 | 11:15 | 12:14 |  |
| 23 | 12 | 11:23 | 16:02 | 4:39 | 12:24 | 13:23 |  |
| 23 | 13 | 11:28 | 16:14 | 4:46 | 12:29 | 13:28 |  |
| 23 | 14 | 11:29 | 16:12 | 4:43 | 12:30 | 13:29 |  |
| 23 | 15 | 11:29 | 16:09 | 4:40 | 12:30 | 13:29 |  |

Table S2: The environmental parameters recorded for cameras of pilot study at Lake Tanganyika, Zambia. Cameras used for comparison between studies are in red font. Rock frequency: the actual count of individual rocks on the examined image. Rock size: Average of estimated rock size according to following categories: 1 = rock size < 1% of image; 2 = rock size < 5% of image; 3 = rock size < 10% of image; 4 = rock size < 25% of image; 5 = rock size > 25% of image.

| **PCT** | **GoPro No** | **Depth**  **(m)** | **Date** | **Visible**  **habitat (%)** | **Sand** | **Vegetation** | **Rock** | **Rock**  **frequency** | **Rock**  **size** | **Habitat type** |
| --- | --- | --- | --- | --- | --- | --- | --- | --- | --- | --- |
| 6 | 1 | 17.9 | 2014-07-27 | 57 | 0.070175439 | 0 | 0.929824561 | 5 | 4 | rock |
| 6 | 2 | 18.4 | 2014-07-27 | 38 | 0 | 0 | 1 | 2 | 5 | rock |
| 6 | 3 | 19.7 | 2014-07-27 | 40 | 1 | 0 | 0 | 0 | 0 | sand |
| 6 | 4 | 20 | 2014-07-27 | 40 | 0.975 | 0 | 0.025 | 1 | 1 | sand |
| 7 | 1 | 11.2 | 2014-07-28 | 75 | 0 | 0 | 1 | 5 | 5 | rock |
| 7 | 2 | 9.8 | 2014-07-28 | 42 | 0 | 0 | 1 | 2 | 4 | rock |
| 7 | 3 | 9 | 2014-07-28 | 34 | 0 | 0 | 1 | 4 | 3 | rock |
| 7 | 4 | 10.5 | 2014-07-28 | 65 | 0.015384615 | 0 | 0.984615385 | 8 | 4 | rock |
| 7 | 5 | 12.3 | 2014-07-28 | 29 | 0 | 0 | 1 | 2 | 4 | rock |
| 8 | 1 | 13.4 | 2014-07-29 | 9 | 0 | 0 | 1 | 3 | 1 | rock |
| 8 | 2 | 14.7 | 2014-07-29 | 18 | 0 | 0 | 1 | 7 | 2 | rock |
| 8 | 3 | 14.1 | 2014-07-29 | 31 | 0.580645161 | 0 | 0.419354839 | 3 | 2 | inter |
| 8 | 4 | 13.1 | 2014-07-29 | 15 | 0 | 0 | 1 | 4 | 2 | rock |
| 10 | 6 | 5 | 2015-07-30 | 50 | 1 | 0 | 0 | 0 | 0 | sand |
| 10 | 7 | 5 | 2015-07-30 | 47 | 1 | 0 | 0 | 0 | 0 | sand |
| 10 | 8 | 4.9 | 2015-07-30 | 45 | 1 | 0 | 0 | 0 | 0 | sand |
| 10 | 9 | 5.3 | 2015-07-30 | 56 | 1 | 0 | 0 | 0 | 0 | sand |
| 10 | 10 | 5.8 | 2015-07-30 | 60 | 1 | 0 | 0 | 0 | 0 | sand |
| 11 | 11 | 5.2 | 2015-07-31 | 48 | 0.25 | 0 | 0.75 | 18 | 1 | rock |
| 11 | 12 | 5.6 | 2015-07-31 | 52 | 0.038461538 | 0 | 0.961538462 | 50 | 1 | rock |
| 11 | 13 | 5.9 | 2015-07-31 | 44 | 0 | 0 | 1 | 40 | 1 | rock |
| 11 | 15 | 6.2 | 2015-07-31 | 57 | 0.157894737 | 0 | 0.842105263 | 20 | 2 | rock |
| 12 | 6 | 9.6 | 2015-07-31 | 51 | 0 | 0 | 1 | 45 | 1 | rock |
| 12 | 7 | 10.2 | 2015-07-31 | 54 | 0.092592593 | 0 | 0.907407407 | 45 | 2 | rock |
| 12 | 8 | 11.4 | 2015-07-31 | 62 | 0 | 0 | 1 | 30 | 3 | rock |
| 12 | 9 | 11.4 | 2015-07-31 | 57 | 0 | 0 | 1 | 16 | 3 | rock |
| 12 | 10 | 10.2 | 2015-07-31 | 53 | 0 | 0 | 1 | 12 | 3 | rock |
| 13 | 16 | 0.5 | 2015-07-31 | 47 | 0 | 0 | 1 | 25 | 2 | rock |
| 13 | 17 | 0.5 | 2015-07-31 | 44 | 0 | 0 | 1 | 20 | 2 | rock |
| 13 | 19 | 0.5 | 2015-07-31 | 34 | 0 | 0 | 1 | 20 | 2 | rock |
| 13 | 20 | 0.5 | 2015-07-31 | 51 | 0 | 0.039215686 | 0.960784314 | 12 | 3 | rock |
| 14 | 11 | 5.5 | 2015-08-01 | 45 | 1 | 0 | 0 | 0 | 0 | sand |
| 14 | 13 | 6.6 | 2015-08-01 | 50 | 0.82 | 0 | 0.18 | 1 | 3 | sand |
| 14 | 14 | 6.5 | 2015-08-01 | 51 | 1 | 0 | 0 | 0 | 0 | sand |
| 14 | 15 | 6 | 2015-08-01 | 60 | 1 | 0 | 0 | 0 | 0 | sand |
| 15 | 6 | 10 | 2015-08-01 | 40 | 1 | 0 | 0 | 0 | 0 | sand |
| 15 | 7 | 10.4 | 2015-08-01 | 46 | 1 | 0 | 0 | 0 | 0 | sand |
| 15 | 8 | 10.6 | 2015-08-01 | 40 | 1 | 0 | 0 | 0 | 0 | sand |
| 15 | 9 | 10.4 | 2015-08-01 | 30 | 1 | 0 | 0 | 0 | 0 | sand |
| 15 | 10 | 10 | 2015-08-01 | 50 | 1 | 0 | 0 | 0 | 0 | sand |
| 16 | 6 | 5 | 2015-08-02 | 44 | 0 | 0 | 1 | 26 | 3 | rock |
| 16 | 7 | 5 | 2015-08-02 | 42 | 0 | 0 | 1 | 30 | 2 | rock |
| 16 | 8 | 5.2 | 2015-08-02 | 66 | 0 | 0 | 1 | 40 | 3 | rock |
| 16 | 9 | 6 | 2015-08-02 | 74 | 0 | 0 | 1 | 30 | 2 | rock |
| 16 | 10 | 6.3 | 2015-08-02 | 40 | 0 | 0 | 1 | 13 | 2 | rock |
| 17 | 11 | 0.5 | 2015-08-02 | 36 | 0 | 0 | 1 | 10 | 3 | rock |
| 17 | 12 | 0.5 | 2015-08-02 | 47 | 0 | 0 | 1 | 35 | 2 | rock |
| 17 | 13 | 0.5 | 2015-08-02 | 50 | 0 | 0 | 1 | 100 | 1 | rock |
| 17 | 14 | 0.5 | 2015-08-02 | 50 | 0 | 0 | 1 | 38 | 2 | rock |
| 17 | 15 | 0.5 | 2015-08-02 | 47 | 0 | 0 | 1 | 9 | 3 | rock |
| 18 | 11 | 0.5 | 2015-08-02 | 57 | 0 | 0 | 1 | 10 | 4 | rock |
| 18 | 12 | 0.5 | 2015-08-02 | 69 | 0 | 0.028985507 | 0.971014493 | 19 | 3 | rock |
| 18 | 13 | 0.5 | 2015-08-02 | 52 | 0 | 0.057692308 | 0.942307692 | 17 | 2 | rock |
| 18 | 14 | 0.5 | 2015-08-02 | 62 | 0 | 0 | 1 | 12 | 4 | rock |
| 18 | 15 | 0.5 | 2015-08-02 | 61 | 0 | 0.016393443 | 0.983606557 | 21 | 3 | rock |
| 19 | 6 | 15 | 2015-08-03 | 40 | 1 | 0 | 0 | 0 | 0 | sand |
| 19 | 7 | 15.1 | 2015-08-03 | 50 | 1 | 0 | 0 | 0 | 0 | sand |
| 19 | 8 | 15.4 | 2015-08-03 | 57 | 1 | 0 | 0 | 0 | 0 | sand |
| 19 | 9 | 15.8 | 2015-08-03 | 58 | 1 | 0 | 0 | 0 | 0 | sand |
| 19 | 10 | 16.4 | 2015-08-03 | 49 | 1 | 0 | 0 | 0 | 0 | sand |
| 20 | 12 | 4.9 | 2015-08-03 | 50 | 1 | 0 | 0 | 0 | 0 | sand |
| 20 | 13 | 5.3 | 2015-08-03 | 53 | 1 | 0 | 0 | 0 | 0 | sand |
| 20 | 14 | 5.4 | 2015-08-03 | 53 | 1 | 0 | 0 | 0 | 0 | sand |
| 20 | 15 | 5.4 | 2015-08-03 | 50 | 1 | 0 | 0 | 0 | 0 | sand |
| 21 | 6 | 20 | 2015-08-05 | 46 | 1 | 0 | 0 | 0 | 0 | sand |
| 21 | 7 | 19.9 | 2015-08-05 | 50 | 1 | 0 | 0 | 0 | 0 | sand |
| 21 | 8 | 19.6 | 2015-08-05 | 47 | 1 | 0 | 0 | 0 | 0 | sand |
| 21 | 9 | 19.6 | 2015-08-05 | 40 | 1 | 0 | 0 | 0 | 0 | sand |
| 21 | 10 | 19.8 | 2015-08-05 | 40 | 1 | 0 | 0 | 0 | 0 | sand |
| 22 | 16 | 10.2 | 2015-08-06 | 31 | 0 | 0 | 1 | 3 | 4 | rock |
| 22 | 17 | 9.9 | 2015-08-06 | 42 | 0.285714286 | 0 | 0.714285714 | 4 | 3 | inter |
| 22 | 18 | 9.7 | 2015-08-06 | 50 | 0 | 0 | 1 | 6 | 3 | rock |
| 22 | 19 | 10.1 | 2015-08-06 | 44 | 0 | 0 | 1 | 2 | 4 | rock |
| 22 | 20 | 10.5 | 2015-08-06 | 41 | 0 | 0 | 1 | 4 | 4 | rock |
| 23 | 12 | 20.9 | 2015-08-07 | 47 | 0 | 0 | 1 | 14 | 3 | rock |
| 23 | 13 | 20.7 | 2015-08-07 | 50 | 0.52 | 0 | 0.48 | 15 | 2 | inter |
| 23 | 14 | 20 | 2015-08-07 | 30 | 0.466666667 | 0 | 0.533333333 | 4 | 2 | inter |
| 23 | 15 | 20 | 2015-08-07 | 35 | 0 | 0 | 1 | 9 | 3 | rock |

Fig S3: 4 Exemplary images from the collection of 28'080 images used in the pilot. Underneath each image the unique ID consisting of PCT, camera and image number (e.g. 016 - 08 - 0021185)


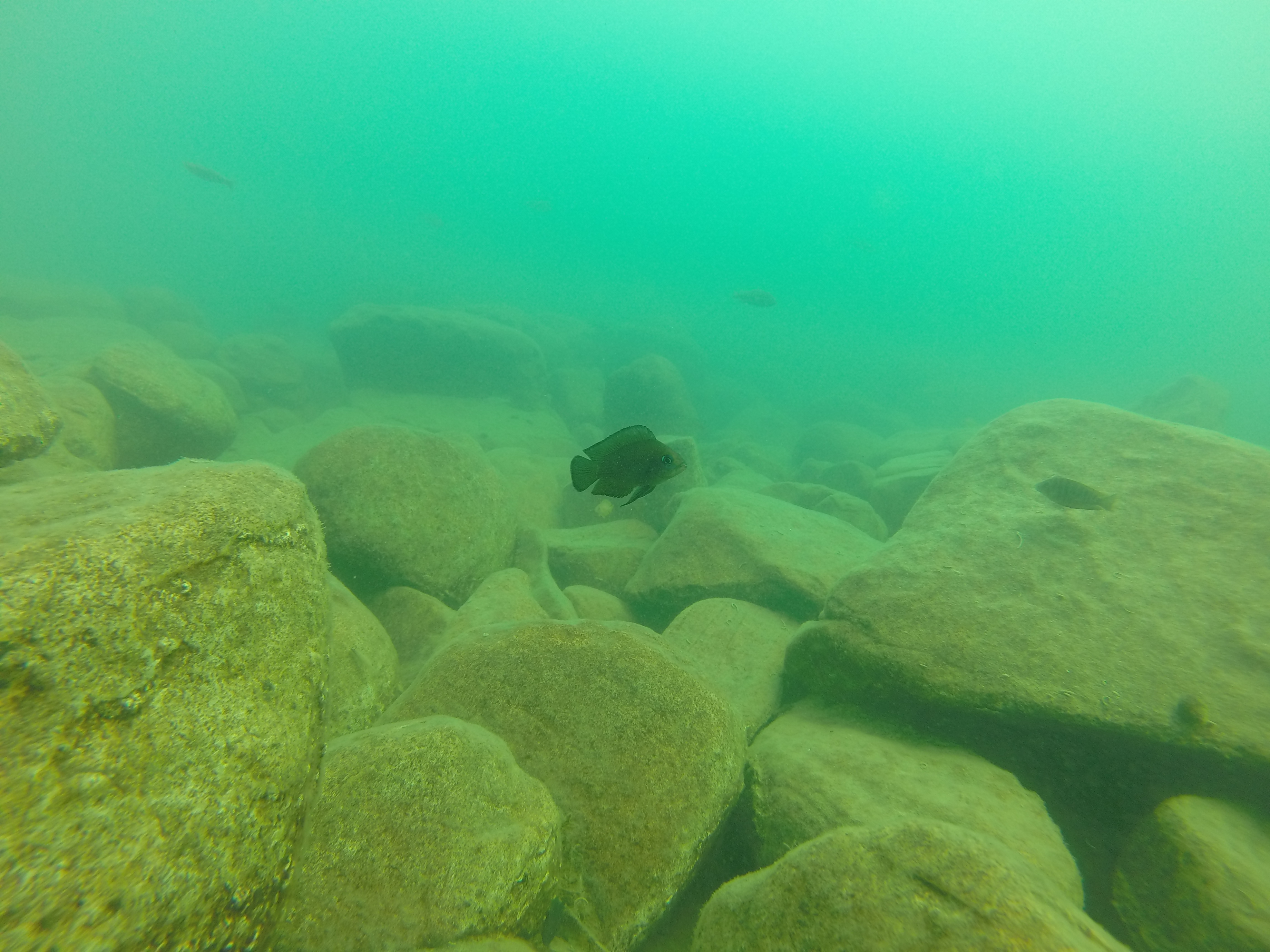
 ID: 016080021185


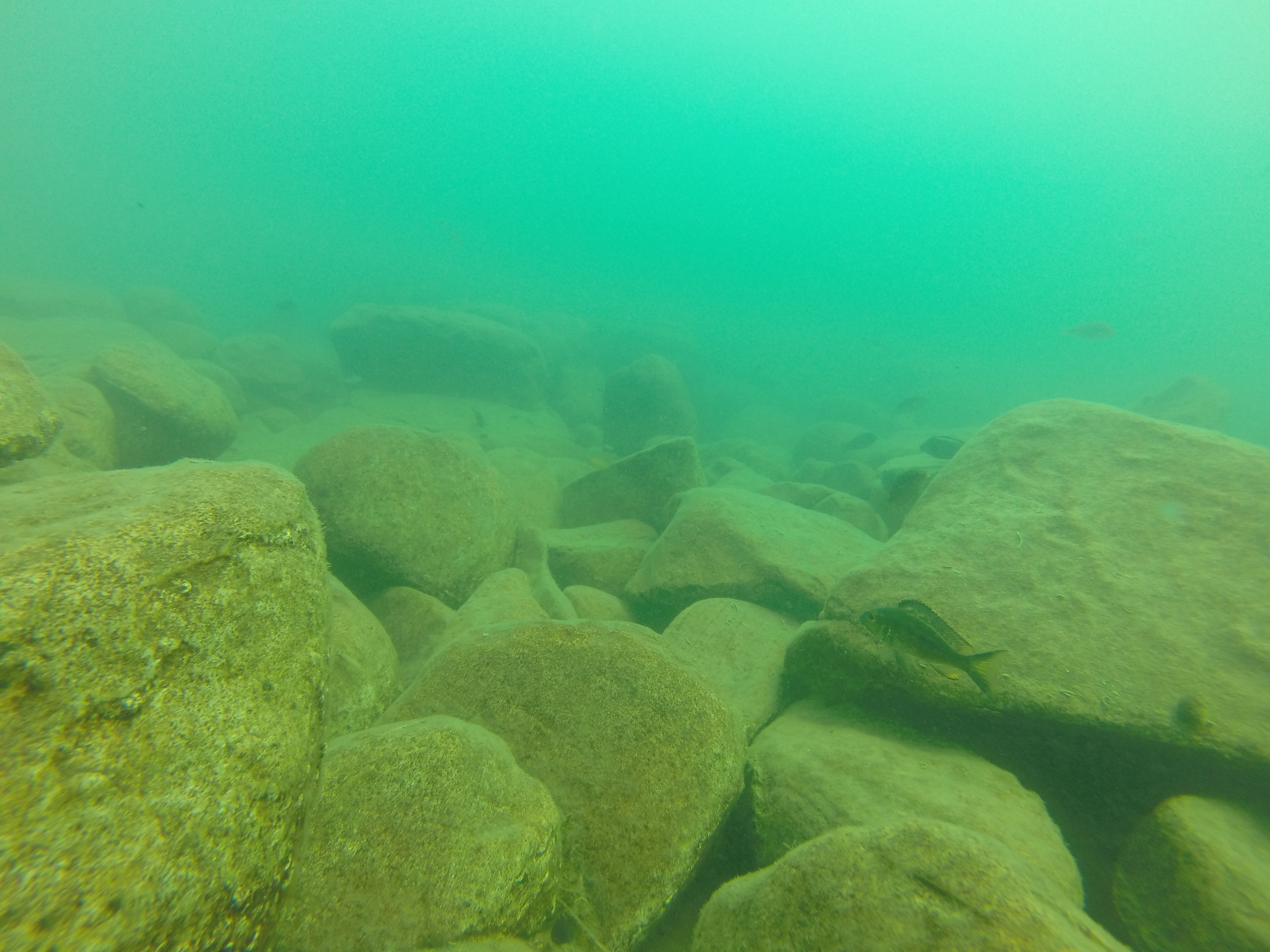


ID: 016080021186


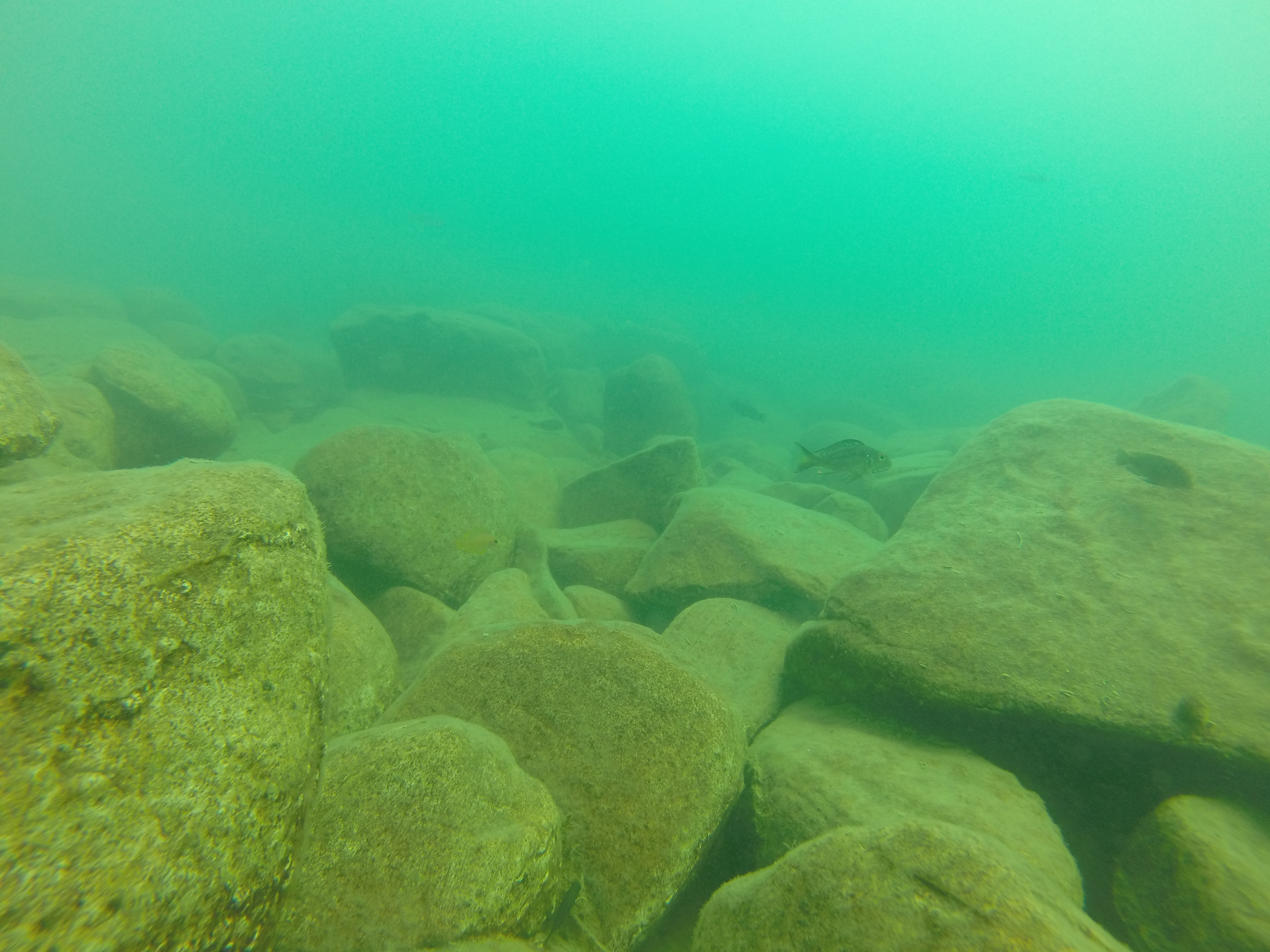


ID: 016080021187


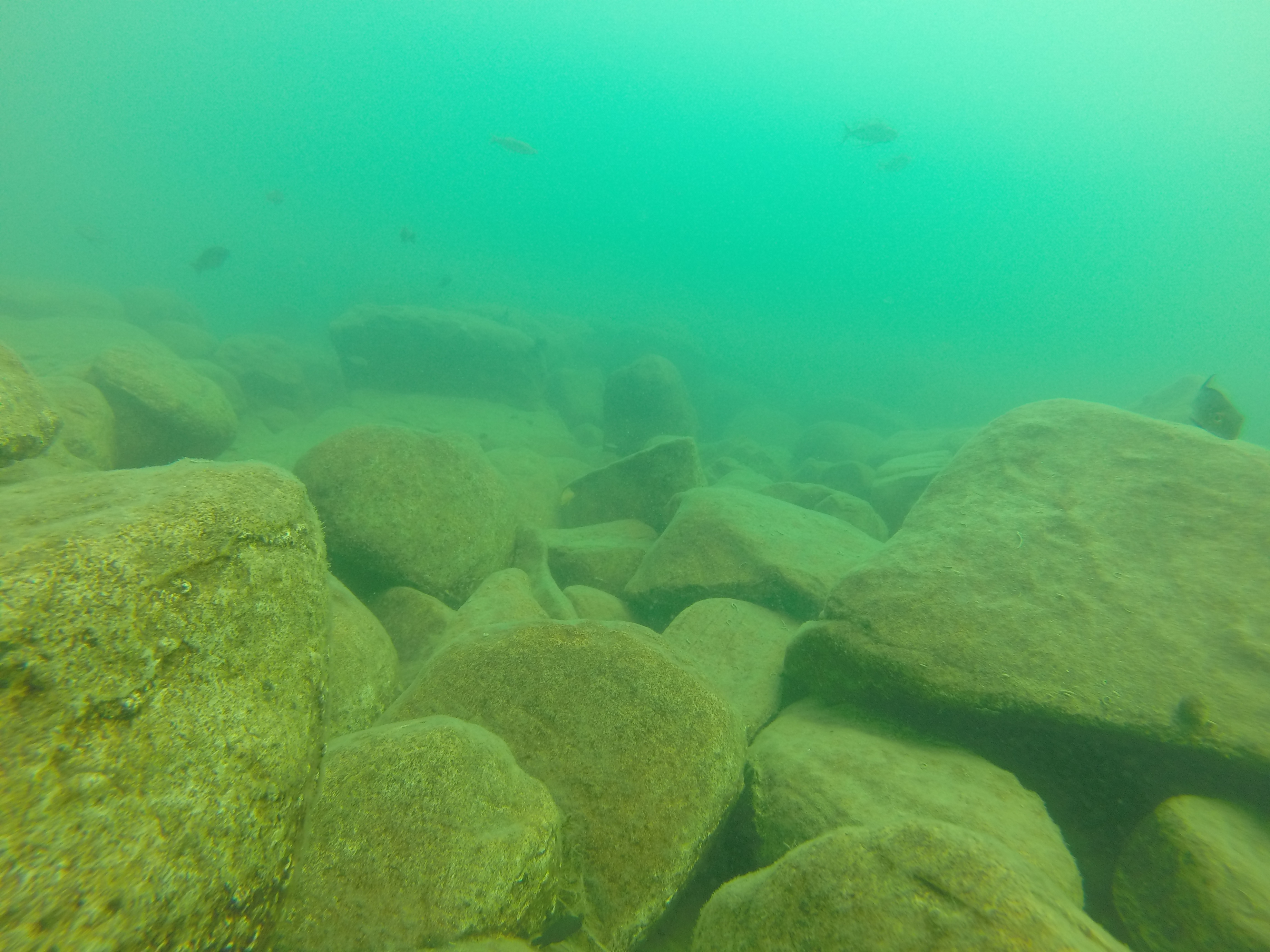


ID: 016080021188

Table S4: List of species accumulation curves (SAC) for cameras and PCT including observed species richness R. R75 = Number of images/PCT to reach 75% of observed R. Saturation = Number of images/PCT to reach plateau of SAC

| **PCT** | **Camera No** | **Observed R** | **R75** | **Saturation** |
| --- | --- | --- | --- | --- |
| 6 | 1 | 9 | 102 | 211.232965 |
| 6 | 2 | 6 | 194 | 409.4361135 |
| 6 | 3 | 4 | 83 | 175.1633099 |
| 6 | 4 | 7 | 144 | 295.9160441 |
| 7 | 1 | 3 | 244 | NA |
| 7 | 2 | 12 | 154 | 350.3111734 |
| 7 | 3 | 8 | 176 | 354.59581 |
| 7 | 4 | 10 | 193 | 398.5763522 |
| 7 | 5 | 2 | 185 | 344.7391987 |
| 8 | 1 | 10 | 180 | 372.349529 |
| 8 | 2 | 13 | 88 | 198.1258269 |
| 8 | 3 | 17 | 176 | 361.8585488 |
| 8 | 4 | 13 | 169 | 350.415613 |
| 10 | 6 | NA | NA | NA |
| 10 | 7 | 2 | 270 | NA |
| 10 | 8 | NA | NA | NA |
| 10 | 9 | NA | NA | NA |
| 10 | 10 | NA | NA | NA |
| 11 | 11 | 19 | 115 | 287.7791974 |
| 11 | 12 | 20 | 60 | 166.7788798 |
| 11 | 13 | 21 | 58 | 161.8040847 |
| 11 | 15 | 14 | 76 | 223.4397398 |
| 12 | 6 | 21 | 144 | 329.972556 |
| 12 | 7 | 22 | 122 | 290.6010749 |
| 12 | 8 | 28 | 159 | 359.189291 |
| 12 | 9 | 22 | 165 | 359.8860764 |
| 12 | 10 | 19 | 87 | 223.2799204 |
| 13 | 16 | 19 | 147 | 339.9163454 |
| 13 | 17 | 17 | 128 | 316.522558 |
| 13 | 19 | 18 | 78 | 217.6308532 |
| 13 | 20 | 16 | 90 | 250.8273168 |
| 14 | 11 | NA | NA | NA |
| 14 | 13 | 2 | 182 | 341.3596037 |
| 14 | 14 | NA | NA | NA |
| 14 | 15 | NA | NA | NA |
| 15 | 6 | NA | NA | NA |
| 15 | 7 | NA | NA | NA |
| 15 | 8 | 2 | 133 | 267.236841 |
| 15 | 9 | 8 | 157 | 312.1597466 |
| 15 | 10 | 7 | 91 | 199.23334 |
| 16 | 6 | 22 | 143 | 320.4703222 |
| 16 | 7 | 19 | 100 | 249.8844949 |
| 16 | 8 | 25 | 110 | 284.5978489 |
| 16 | 9 | 22 | 126 | 382.1523957 |
| 16 | 10 | 17 | 133 | 278.9561594 |
| 17 | 11 | 7 | 66 | 180.9234464 |
| 17 | 12 | 15 | 144 | 324.8903357 |
| 17 | 13 | 9 | 32 | 119.9485596 |
| 17 | 14 | 11 | 68 | 171.2521393 |
| 17 | 15 | 9 | 143 | 321.8236654 |
| 18 | 11 | 11 | 157 | 378.2470096 |
| 18 | 12 | 16 | 100 | 263.5366491 |
| 18 | 13 | 15 | 57 | 147.5909741 |
| 18 | 14 | 10 | 42 | 115.8806789 |
| 18 | 15 | 17 | 74 | 244.5771509 |
| 19 | 6 | 2 | 47 | 106.5898097 |
| 19 | 7 | 3 | 103 | 216.9687519 |
| 19 | 8 | 5 | 184 | 350.6502739 |
| 19 | 9 | 8 | 157 | 314.4227015 |
| 19 | 10 | 12 | 195 | 388.8472455 |
| 20 | 12 | NA | NA | NA |
| 20 | 13 | NA | NA | NA |
| 20 | 14 | NA | NA | NA |
| 20 | 15 | NA | NA | NA |
| 21 | 6 | NA | NA | NA |
| 21 | 7 | 7 | 54 | 127.1138529 |
| 21 | 8 | 10 | 87 | 204.7829463 |
| 21 | 9 | 8 | 144 | 308.7899662 |
| 21 | 10 | 7 | 103 | 229.573169 |
| 22 | 16 | 20 | 133 | 282.439628 |
| 22 | 17 | 11 | 164 | 343.0628605 |
| 22 | 18 | 16 | 113 | 275.178596 |
| 22 | 19 | 12 | 73 | 166.6120878 |
| 22 | 20 | 23 | 156 | 366.9119918 |
| 23 | 12 | 14 | 172 | 363.4719851 |
| 23 | 13 | 18 | 128 | 270.8745324 |
| 23 | 14 | 9 | 175 | 357.8284875 |
| 23 | 15 | 15 | 173 | 353.1349722 |
| **PCT** |  | **Observed R** | **R75** | **Saturation** |
| 6 |  | 14 | 3 | NA |
| 7 |  | 18 | 3 | 9.825 |
| 8 |  | 22 | 2 | 6.12222222 |
| 12 |  | 33 | 2 | 5.04214876 |
| 15 |  | 9 | 3 | 7.172 |
| 16 |  | 34 | 2 | 5.79278351 |
| 19 |  | 15 | 3 | 7.28974359 |
| 21 |  | 13 | 3 | 5.772 |
| 10 |  | 1 | 1 | NA |
| 11 |  | 25 | 2 | 4.13829787 |
| 14 |  | 3 | 2 | NA |
| 17 |  | 13 | 2 | 5.884 |
| 18 |  | 19 | 2 | 5.3745098 |
| 20 |  | 1 | 1 | NA |
| 23 |  | 23 | 2 | 4.03333333 |
| 13 |  | 20 | 1 | 4.20909091 |
| 22 |  | 28 | 2 | 5.36168224 |

Table S5: 1’000 random subsamples of 12 images, comparing analysed hour (group 2) and images from starting point until selection (group 1). ns = non-significant; s = significant, p-value = p-value at 95% confidence. Tests were performed on raw count data and number of species for subsamples between first and second part and within second part.

|  | **COUNT DATA** | | | | | | **SPECIES DATA** | | | | | |
| --- | --- | --- | --- | --- | --- | --- | --- | --- | --- | --- | --- | --- |
|  | between group 1 & 2 | | | within group 2 | | | between group 1 & 2 | | | within group 2 | | |
| PCT | **ns** | **s** | p-value | **ns** | **s** | p-value | **ns** | **s** | p-value | **ns** | **s** | p-value |
| 40 \| 28 | 975 | 25 | 0.08 | 999 | 1 | 0.27 | 989 | 11 | 0.13 | 999 | 1 | 0.38 |
| 35 \| 07 | 937 | 63 | 0.04 | 800 | 200 | 0.00 | 990 | 10 | 0.14 | 998 | 2 | 0.18 |

Fig S6: Boxplot of comparison among cichlid density for the pilot and the three comparative studies. Densities calculated for species with 4 or more counts for the area of observation: This study (125 m2), MetA (400 m2), MetB (180 m2), MetC (1'200 m2) (***: P = 0.00, ns: not significant).

Fig S7: Comparison between the observed number of individuals of large and small cichlid species, presented in separate boxplots for each study. All pairs were tested using Mann-Whitney U test and proved not significant: This study, W = 186, P = 0.44; MetA, W = 182, P = 0.83; MetB, W = 255,
P = 0.56; MetC, W = 89, P = 0.56.


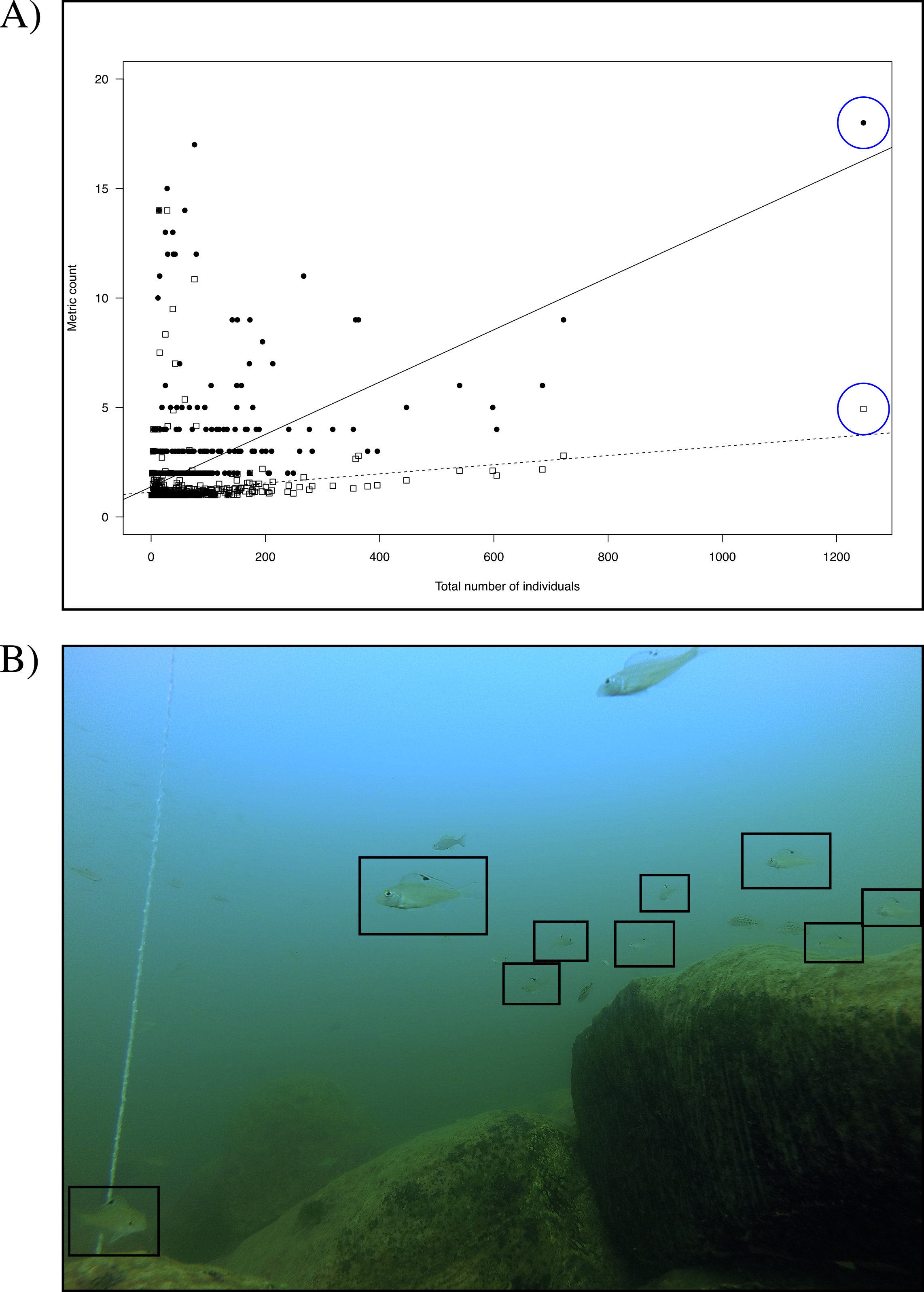


Fig S8: A) The two metrics, MaxN (•) and mean (☐) against the total number of individuals per species per camera. The mean underestimates more "densely" occurring species at a site, e.g. *Xenotilpia spiloptera* (blue circle), as well as species with only few occurrences (in which the mean value is almost always close to 1). B) Example of an image showing 9 recorded specimens of *X. spiloptera* (squares on the image). The other individuals were discarded on the basis of our "Identification and count protocol" (Table 1). For this species, the mean for the respective camera was calculated at 4.4 specimens (due to fewer sightings on other images). However, a maximum of 18 specimens was observed on one image, suggesting that MaxN more accurately represents the number of individuals present at any given location.

Fig S9: Total sum of 17 PCT fish counts at pilot location. Cichlids divided into three benthic-pelagic categories as defined by Colombo, Indermauer, Meyer & Salzburger (2016)
